# Genetic diversity in developmental responses to light spectral quality in barley (*Hordeum vulgare* L.)

**DOI:** 10.1186/s12870-020-02416-1

**Published:** 2020-05-12

**Authors:** Arantxa Monteagudo, Tibor Kiss, Marianna Mayer, Ana M. Casas, Ernesto Igartua, Ildikó Karsai

**Affiliations:** 1 Aula Dei Experimental Station (EEAD-CSIC), Avda. Montañana 1005, E-50059 Zaragoza, Spain; 2 Centre for Agriculture Research (ATK), Martonvásár, H-2462 Hungary

**Keywords:** Barley, Flowering, Gene expression, Growth, Light quality

## Abstract

**Background:**

Plants use light wavelength, intensity, direction and duration to predict imminent seasonal changes and to determine when to initiate physiological and developmental processes. Among them, crop responses to light are not fully understood. Here, we study how light quality affects barley development, using two broad-spectrum light sources, metal halide (M) and fluorescent (F) lamps. Eleven varieties with known allelic variants for the major flowering time genes were evaluated under controlled conditions (long days, same light intensity). Two experiments were carried out with fully-vernalized plants: 1) control treatments (M, F); 2) shifting chambers 10 days after the start of the experiment (MF, FM).

**Results:**

In general, varieties developed faster under longer exposure to M conditions. The greatest differences were due to a delay promoted by F light bulbs, especially in the time to first node appearance and until the onset of stem elongation. Yield related-traits as the number of seeds were also affected by the conditions experienced. However, not each variety responded equally, and they could be classified in insensitive and sensitive to light quality. Expression levels of flowering time genes *HvVRN1*, *HvFT1* and *PPD-H1* were high in M, while *HvFT3* and *HvVRN2* were higher under F conditions. The expression under shift treatments revealed also a high correlation between *HvVRN1* and *PPD-H1* transcript levels.

**Conclusions:**

The characterization of light quality effects has highlighted the important influence of the spectrum on early developmental stages, affecting the moment of onset of stem elongation, and further consequences on the morphology of the plant and yield components. We suggest that light spectra control the vernalization and photoperiod genes probably through the regulation of upstream elements of signalling pathways. The players behind the different responses to light spectra found deserve further research, which could help to optimize breeding strategies.

## Background

As plants are sessile organisms, they measure quantity (intensity), quality (spectral composition), direction and duration (photoperiod) of light to regulate their development and to acclimatise to the surrounding environment [1–3]. The integration of several cues is essential for the plant to decipher what is happening around it and respond accordingly. In an example exposed by Casal and Qüesta [4]: “the same photoperiod can take place in late summer and spring”, thus an unique signal does not provide enough information, and more signals are needed to solve that ambiguity, similarly to what occurs in winter crops, in which the memory of winter temperatures complements photoperiodic information to define the season. Spectral composition is a parameter of light sensed by plants that vary with altitude, latitude, seasons, and climatic and atmospheric factors [5]. During the day, spectral energy distribution of solar light changes between day, dawn and dusk, thus light quality contributes to determine the precise timing of photoperiod signals. Also, the relative levels of blue, red and far-red wavelengths change in many circumstances, for instance in presence of clouds. Thus, natural light spectra vary in a continuous state of flux, being accompanied by other environmental changes [6]. Finally, the radiation that reaches a plant is affected by these factors and by the canopy of neighbours in the vicinity [7]. All these circumstances cause spectral differences that lead to complex interactions, affecting plant growth [8].

Flowering is a complex process that involves signals from several pathways recording information from the plant and the environment, and promoting transition to the reproductive stage under favourable conditions. Barley (*Hordeum vulgare* L.) and wheat (*Triticum* spp.) are long-day (LD) plants, flowering earlier under increasing daylengths. Flowering promotion is controlled through the interplay of the major flowering time genes, *VERNALIZATION 1* (*HvVRN1)*, *VERNALIZATION 2* (*HvVRN2*) and the homologous of *FLOWERING LOCUS T* (*FT*), *HvFT1*. Vernalization and photoperiod signals must be integrated to allow timely flowering; thus, the aforementioned genes interplay with the long and short photoperiod response genes, *PHOTOPERIOD RESPONSE1* (*PPD-H1*, whose candidate is *HvPRR37* [9]), and *PHOTOPERIOD RESPONSE2* (*PPD-H2*, whose candidate is *HvFT3* [10]), respectively.

The effect of light quality in plant growth has been widely studied, especially in horticultural and ornamental species. Nowadays, new protocols for growing crops as fast as possible in indoor facilities are at the cutting edge of plant breeding research [11]. Optimizing the spectral composition of the growth chambers must be considered when planning an experiment, to favour fast development or facilitating replication of the results. In a recent experiment with LED lighting in wheat [3], light spectra and intensity affected development, metabolism, yield and quality, opening the possibility to modulate light to improve them.

The red to far-red (R:FR) ratio has been extensively studied due to its relation with vegetation shade and its consequent involvement in shade avoidance syndrome, including comparisons of different light sources [12–14]. Lately, much attention has been given to other waveband ratios to which plant responsiveness is also highly dependent on the species and cultivar of interest [15]. In wheat, for instance, light quality was determinant in stem elongation, which was influenced by blue, green and far-red light antagonistically [3].

Our work has focused on the effect on plant development of two conventional artificial lighting regimens that provide different spectral quality: fluorescent (F) and metal halide (M) bulbs. Our aims are 1) to characterize the effect of different lighting systems and examine the consequences of transferring plants between both conditions, 2) to evaluate natural variation in the responses of a set of varieties from different origins, and 3) to determine the molecular effect on vernalization and photoperiod pathways at early developmental phases. We have compared the responses on morphology and development, including apex examination, and gene expression studies of major flowering time genes in barley.

## Results

### Developmental responses to light quality

In this work, the effect of two broad-spectrum light sources was investigated: fluorescent (F) and metal halide (M) light bulbs. Both light sources emit continuous visible spectra with major differences in specific regions (Figure S1). In general, fluorescent light was rich in the green-yellow (GY) and red (R) regions, whereas metal halide spectrum was more balanced across the photosynthetically active region (PAR).

**Fig. 1:**
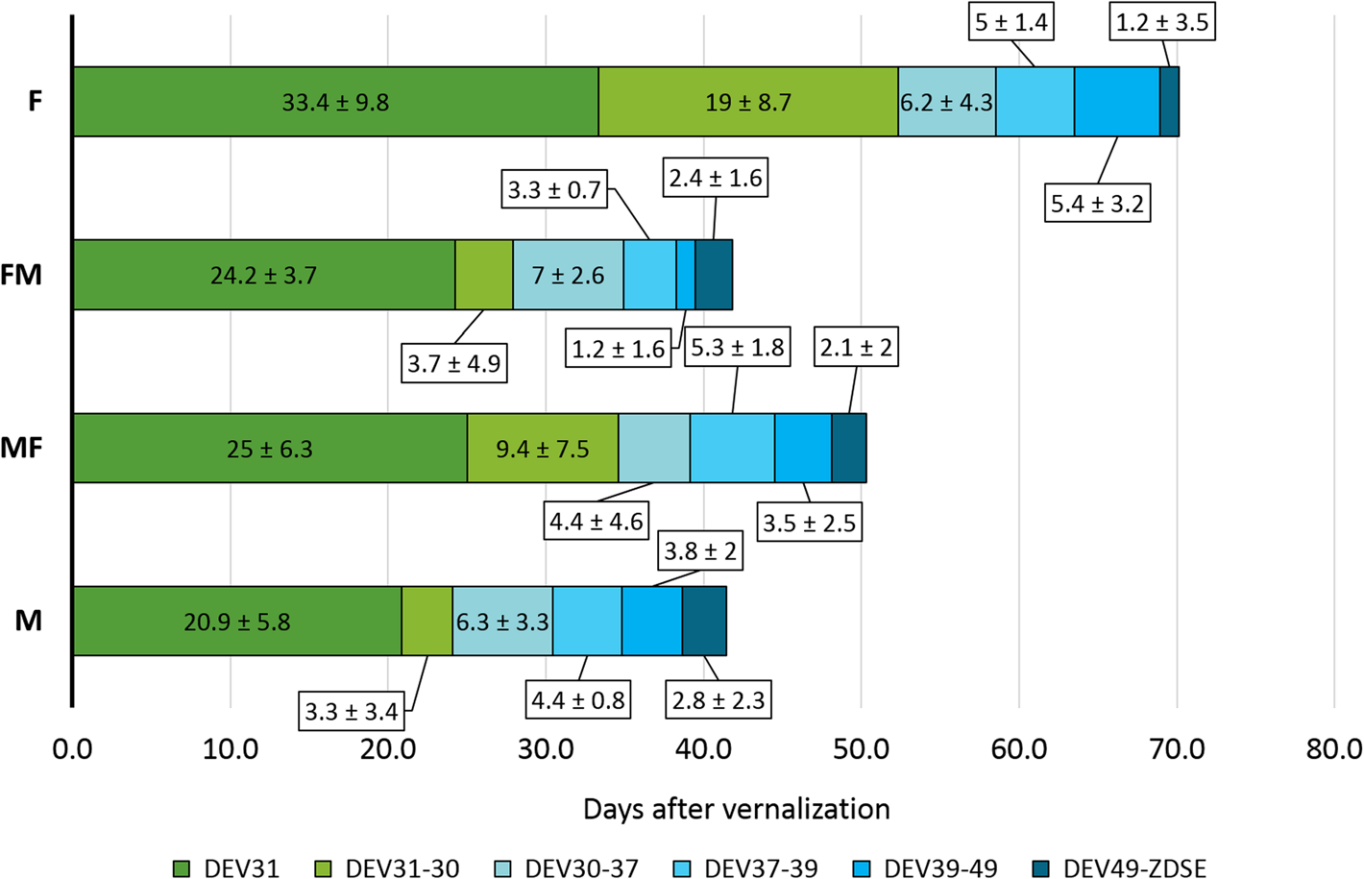
Duration of the different developmental phases under different lighting treatments. Metal halide (M), 10 days in fluorescent and then shift to metal halide (FM); fluorescent (F) and 10 days in metal halide and then shift to fluorescent (MF) bulbs. Mean of 11 varieties and 4 replicates per variety (*n* = 44), and standard deviation are represented as numbers in each phase. DEV31, first node appearance; DEV31–30, days from first node appearance to the onset of stem elongation; DEV30–37, days from the onset of stem elongation to the appearance of the flag leaf; DEV37–39, days to complete expansion of the flag leaf; DEV39-DEV49, days from the completion of the flag leaf to awns appearance; DEV49-ZDSE, days from awns appearance to the end of stem elongation

Eleven barley varieties with different allelic composition at *HvVRN1*, *HvVRN2*, *HvFT1* and *HvFT3* genes (described in Table 1) were evaluated under M and F conditions, and in shift treatments that involved the interchange between growth chambers 10 days after the end of the vernalization process. In general, barley plants grown under F lighting flowered later than in M conditions (Fig. 1, M and F). Among the phenological phases encompassing the flowering process (phenophases), time to first node appearance (DEV31) and from there until the onset of stem elongation phase (DEV30) were the most affected by the lighting conditions (much longer in F).

**Table 1:** List of the barley genotypes examined and allelic variants for the major flowering time genes

Variety	Origin	Row number	Growth habit^a^	***HvVRN1***^b^	***HvVRN2***^c^	***HvFT1***^d^	***PPD-H1***^e^	***HvFT3***^f^
Kold	USA	6	W	vrn1	VRN2	AG	PPD1	ppd2
Price	USA	6	W	vrn1	VRN2	TC	PPD1	PPD2
WA1614–95	USA	6	F	vrn1	vrn2	AG	PPD1	ppd2
Haruna Nijo	Japan	2	F	vrn1	vrn2	TC	PPD1	PPD2
Eight-Twelve	USA	6	W	vrn1	VRN2	AG	PPD1	PPD2
Scio	USA	6	F	vrn1	vrn2	AG	PPD1	PPD2
Dicktoo	USA	6	F	vrn1	vrn2	TC	PPD1	ppd2
Ragusa	Croatia	6	F	VRN1–6	vrn2	AG	PPD1	ppd2
Esterel	France	6	W	vrn1	VRN2	TC	PPD1	ppd2
SBCC016	Spain	6	W	VRN1–6	VRN2	AG	PPD1	PPD2
SBCC046	Spain	6	W	VRN1–6	VRN2	AG	PPD1	ppd2

^a^ Growth habit deduced from the alleles at loci *HvVRN1* and *HvVRN2*. W, winter; F, facultative

^b^ Alleles based on the size of intron 1 [16]

^c^ Presence/absence of *HvZCCT* [17]

^d^ Alleles based on two SNPs in intron 1 [18]

^e^ Alleles based on SNP22 [9]

^f^ Presence/absence of *PPD-H2* [10]

Regarding the shift treatments, in general, plants transferred from M to F (MF) took longer to reach the end of the stem elongation (ZDSE) than plants transferred from F to M (FM), but developed earlier than those in F conditions (Fig. 1). In both shifts, plants showed similar average duration of DEV31 phase, which took around 25 days. However, from that moment on, FM conditions shortened the duration of the different phenophases, becoming more similar to development in M conditions. Overall, the differences in phase duration among treatments were more concentrated in two stages, before and after onset of stem elongation (DEV30). The total duration until DEV30 was 24.2, 27.9, 34.4 and 52.4 days (at M, FM, MF, F, respectively). After DEV30, the sum showed little variation among treatments, being 13.9, 15.3, 17.3 and 17.8 days, at FM, MF, M and F, respectively. The analyses of variance revealed significant differences between treatments, varieties and the interaction treatment × variety for all the developmental phases (Figure S2).

Treatments M and FM showed rather similar duration of the phenophases (Fig. 1). Ten days in fluorescent conditions caused slight delay in occurrence of DEV31 and DEV30 (later FM), but this delay was almost completely offset by the hastening of the three last phases. Larger differences were found when comparing F and MF. Most of the phenophases were shorter after a period of 10 days under M conditions (Fig. 1), with the largest differences in the first two phases (DEV31 and DEV31–30). A brief exposition of 10 days under metal halide lighting (MF) supposed an acceleration of DEV31 up to 17.5 days, and an acceleration of awns appearance (DEV49) up to 31 days (Figure S3).

Plants grown in continuous F light had a higher final number of leaves in the main stem than the other three treatments. Phyllochrons were also affected by the light treatments, with significantly longer phyllochron the more the plants were under F light (Figure S4).

The varieties showed different patterns of development in the control (M) conditions (Figure S5). Some varieties reached DEV31 at the same time despite the lighting system used, whereas fluorescent light delayed the transition in other cases, as can be noticed by the distance from the dots to the 1:1 line. A diversity of responses was observed, with varieties Haruna Nijo and Kold as the most stable across light treatments.

### Dynamics of apices and plant height

**Fig. 2:**
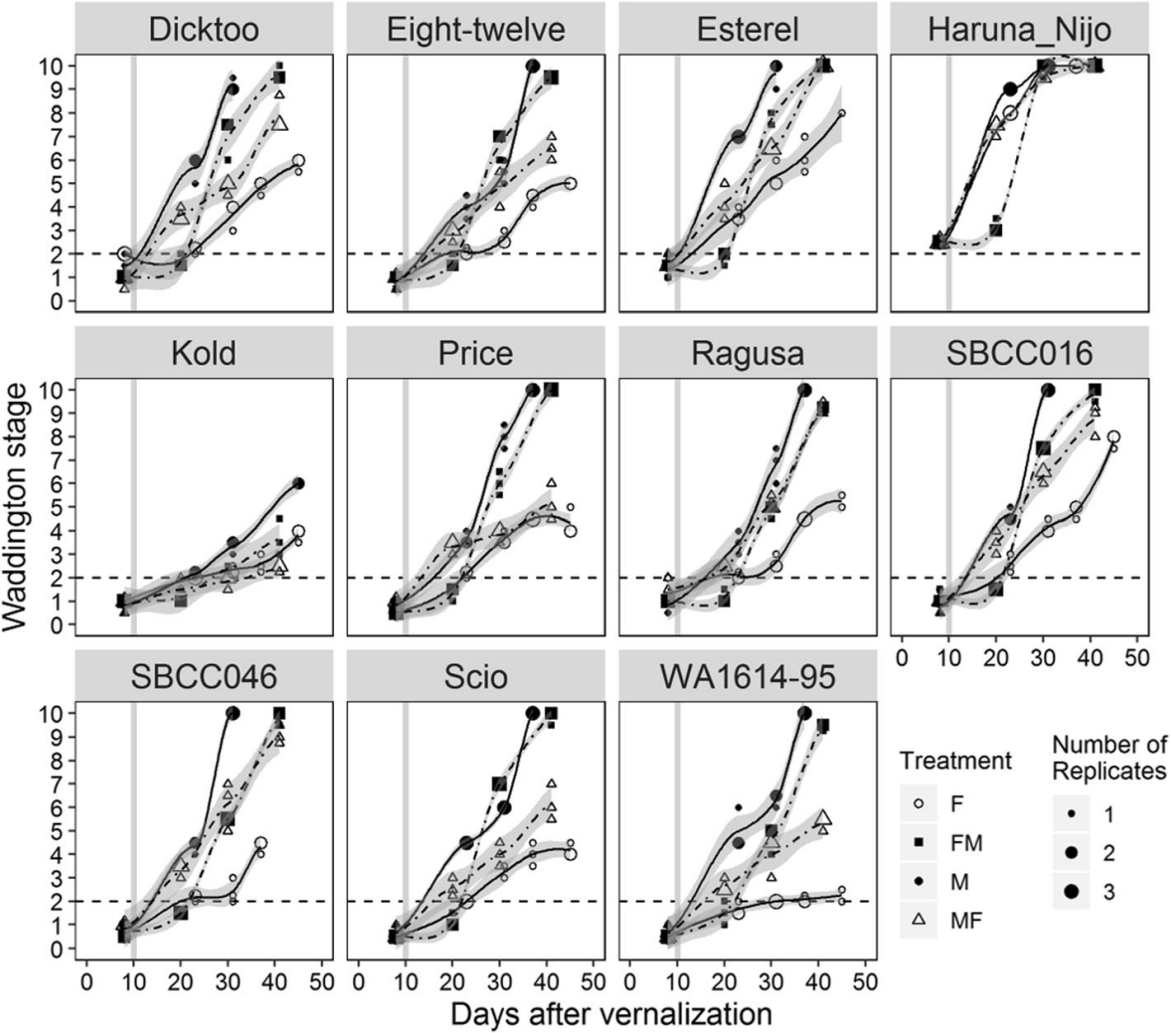
Dynamics of apex development under different light quality conditions. Each block represents a variety. Solid lines denote control treatments (white point is fluorescent, black point is metal halide), and dashed lines denote shift treatments (FM in squares, MF in triangles). The size of each dot represents the number of apices (biological replicates) at that Waddington stage. Dashed horizontal lines mark WD2, the double ridge stage, considered as transition from vegetative to reproductive phase. Vertical solid grey line denotes the shift day: the day when light quality treatments were changed. The shaded area indicates the 95% confidence interval (loess smooth line) calculated using a polynomial regression model

Dissection of plants was carried out at different time points of the experiment to study the development of the apices. Waddington stage and apex length were recorded. Apex development was delayed in plants growing under fluorescent conditions (Fig. 2). For apices dissected 10 days after the shift (20 days after vernalization), two contrasting patterns were detected: shifted plants in FM showed the same delayed pattern as the plants from the initial F chamber but plants in MF progressed as readily as plants in M. Thus, the initial period under metal halide light produced a developmental boost that accelerated development even after the shift to fluorescent lighting conditions. From 10 days after the shift to the last apex dissection, we observed different patterns of responses: (A) plants that adapted to the new conditions, showing the expected pattern of growth according to the new conditions (Scio and Price), and (B) plants which showed small or no differences between treatments (Kold, Haruna Nijo, Ragusa).

The development of the apices in the shift treatments showed a rapid morphological change, whereas apex length presented a slower response (Figure S6). Apex development was measured several times during the experiment, but only once on the same date for all four treatments (day 30 after the start of the experiment, 20 days after the shifts). At this date, there were significant differences for apex development among varieties, treatments and their interaction (Table S1).

**Fig. 3:**
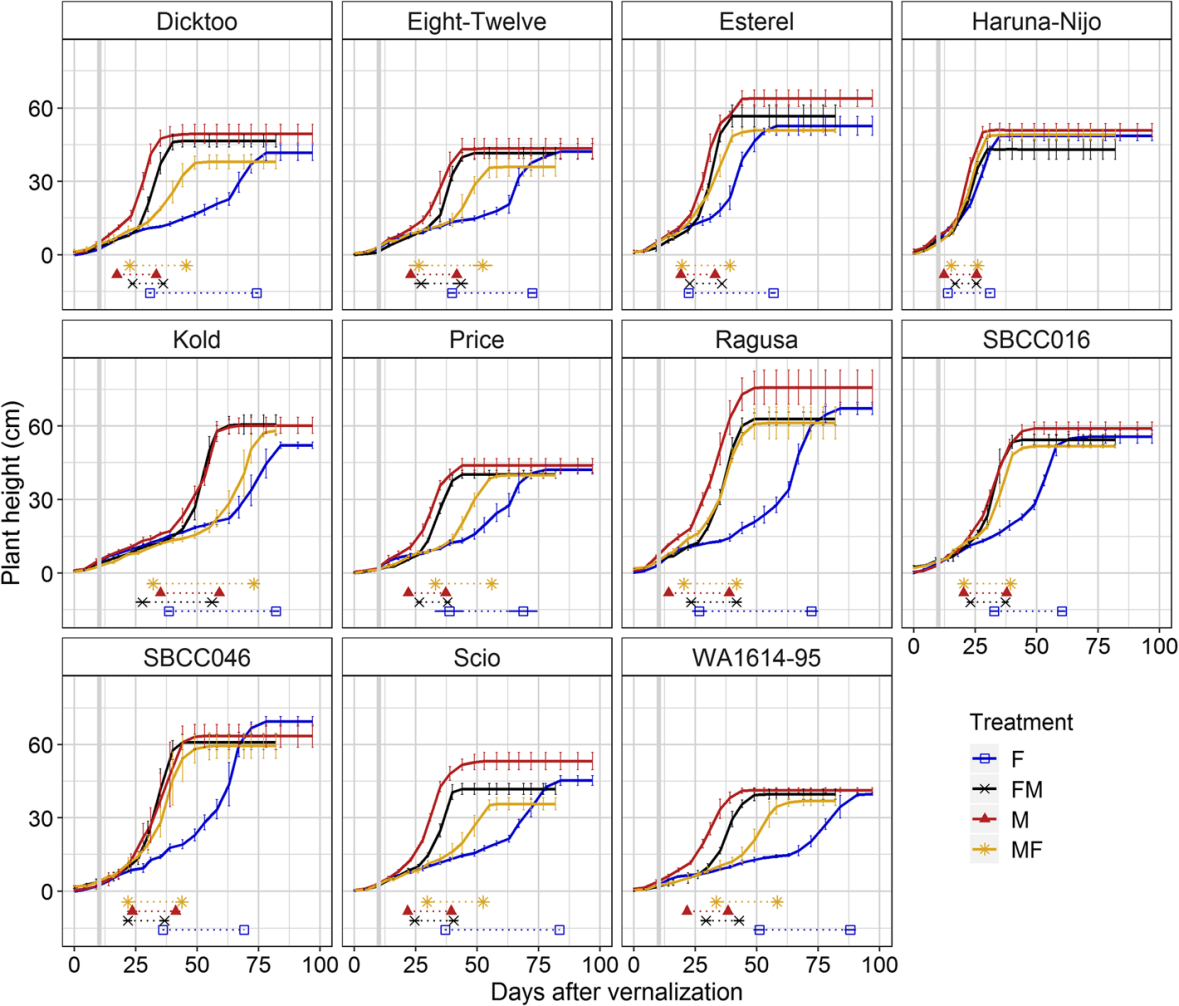
Dynamics of plant height under different light quality conditions. Each block represents a variety. Blue line, fluorescent (F); black line, FM; red line, metallic (M); yellow line, MF. Each line represents the average of 4 biological replicates, and bars denote standard deviation. Under the curves, horizontal lines represent the time from first node appearance (DEV31, first dot) to awns appearance (DEV49) in the main stem. Vertical solid grey lines denote the shift day (the day when plants were switched between growth chambers with different light quality treatment)

We observed diversity in the dynamics of plant height in response to light quality (Fig. 3). Some varieties showed a different behaviour only in F (Esterel, Ragusa, SBCC016 and SBCC046). Other varieties showed different development under the four treatments, with plants reaching the onset of stem elongation first under M, then in FM and MF, and finally in F conditions (Dicktoo, Eight-Twelve, Price, Scio and WA1614–95).

Final plant height was affected by the treatments (Figure S4), but less than other traits like DEV30 (Figure S2) or the days to reach the 50% of the final plant height (Figure S4).

We analysed whether genotypic groups based on allelic composition at the flowering time genes described in Table 1 had any impact on development (Table S2). We found only minor differences at the variety by treatment interaction level (*HvFT1* by treatment for final plant height).

### Reproductive fitness traits

**Fig. 4:**
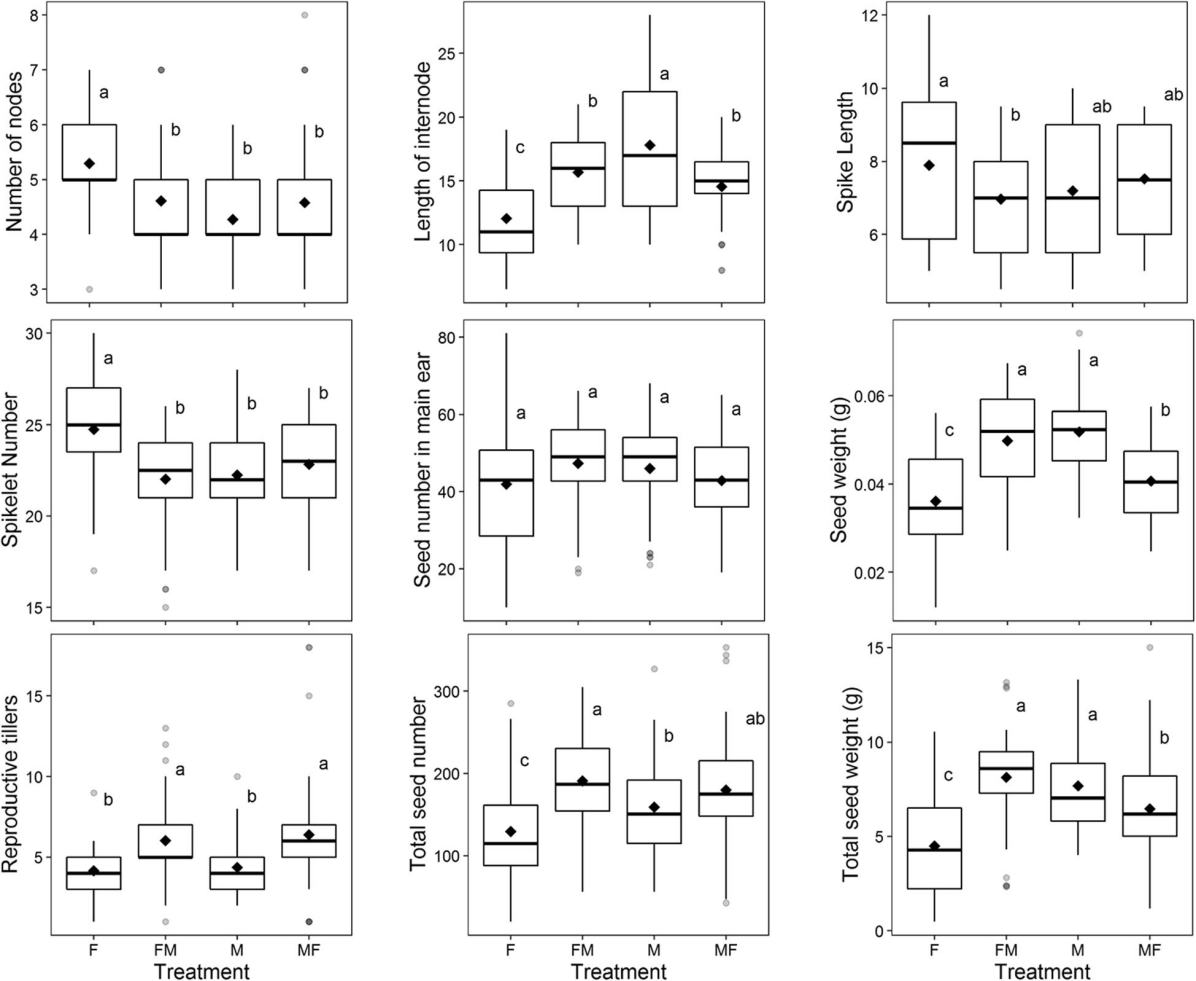
Boxplots of traits measured at harvest in the four light quality treatments. F, fluorescent light; FM, 10 days in fluorescent light, then shift to metal halide light; M, metal halide light; MF, 10 days in metal halide, then shift to fluorescent conditions. In each box plot (n = 44), the mean is represented with a black diamond; the horizontal bar splitting the box represents the median; the height of the box represents the interquartile range, and the whiskers length represent 1.5 times of the interquartile range. From top to bottom, and left to right: number of nodes in the main stem; length of last internode of main stem in cm; length of the main spike in cm; number of spikelets per main spike; number of seeds in the main spike; individual seed weight (g) in the main spike; number of reproductive tillers per plant; total number of seeds per plant; and total seed weight per plant. Results derived from 4 plants per variety, 11 varieties. Different letters represent significant differences between treatments in a post-hoc LSD-test, for a *P*-value < 0.05

The different duration of the developmental phases affected the traits measured at harvest. Plants grown under fluorescent conditions showed more nodes, shorter last internode, more spikelets per spike, less and lighter seeds (Fig. 4) and more final leaves (Figure S7) than other treatments. Results showed differences between treatments for all the traits, except for the number of seeds in the main spike (ANOVA *p*-value = 0.182). Plants grown in metal halide conditions had heavier seeds than plants grown under fluorescent conditions. Plants grown in FM showed more reproductive tillers and number of seeds than those in M. Under fluorescent light, plants had more tillers in nine out of eleven varieties (Figure S8), but many did not produce spikes, resulting in the same number of reproductive tillers as in metal halide (Fig. 4).

### Gene expression

**Fig. 5:**
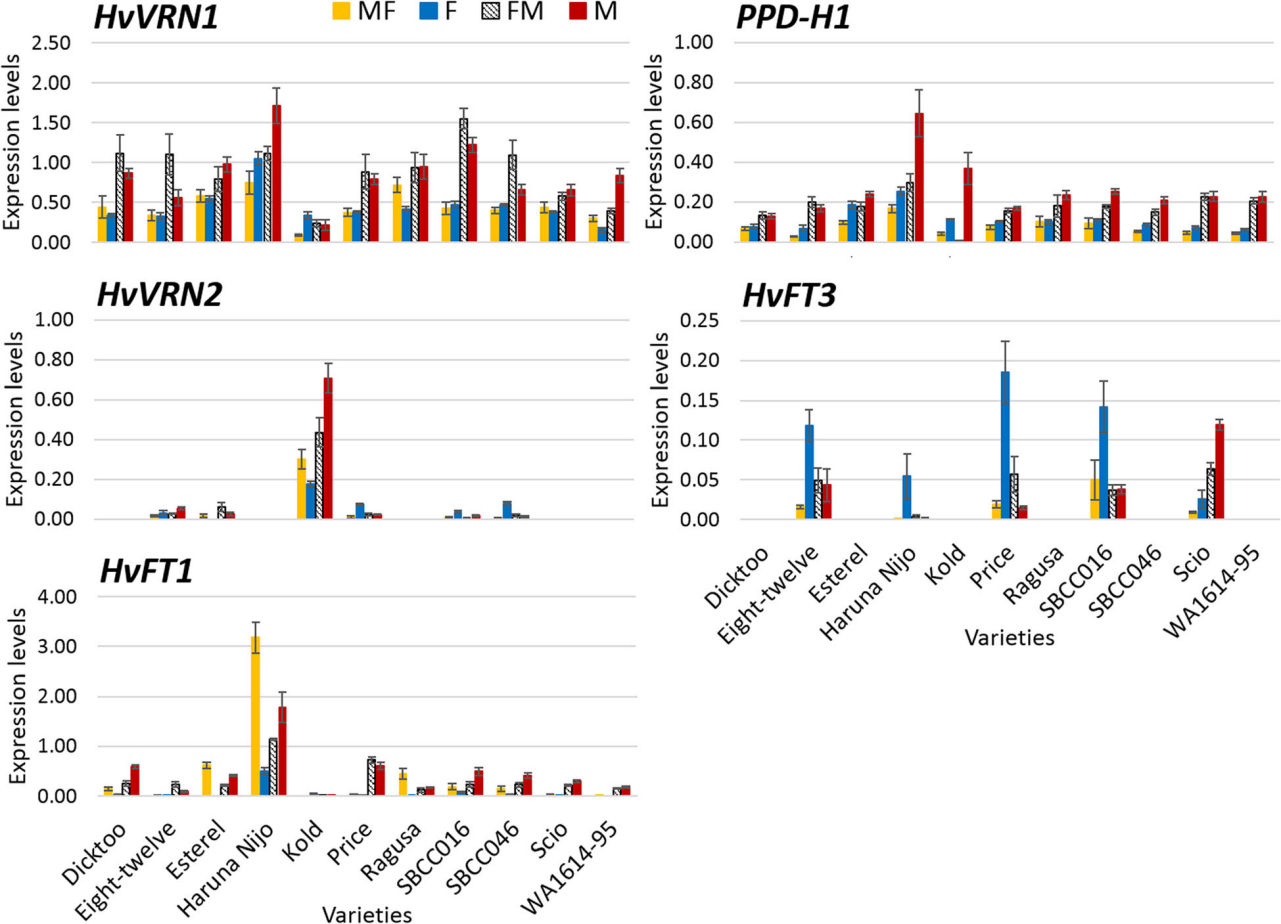
Gene expression under the different treatments. Samples were taken 20 days after the end of the vernalization treatment. Gene expression relative to Actin. Mean of 3 biological replicates. Bars represent SEM

Expression of the major flowering time genes was analysed in leaves of plants 20 days after the end of the vernalization treatment in all the conditions (10 days after the shift in FM and MF, Fig. 5, Table S3). For all varieties, except the earliest Haruna Nijo, sampling time occurred when the apices of most genotypes already had undergone the vegetative-generative transition, WD2.0 (Fig. 2) and the plants were either before DEV31, or between DEV31 and DEV30 (Figure S3).

Overall, *HvFT1* was most expressed in Haruna Nijo, the earliest variety in this study. On the other hand, Kold, the latest variety, expressed *HvVRN2* under all treatments, whereas its expression was much lower in other genotypes. *HvFT3*, when the functional variant was present, was upregulated under fluorescent condition in most cases (Scio was the exception). Both genes were expressed in that condition.

An effect of light quality on *HvVRN1* and *PPD-H1* was observed. Both showed higher expression under M and FM conditions. These genes showed a rapid adaptation to new conditions, as their expression levels were more similar between MF and F, and between FM and M conditions (Fig. 5). Interestingly, the expression of *PPD-H1* and *HvVRN1* showed a moderately high and positive Pearson correlation (r = 0.59).

**Fig. 6:**
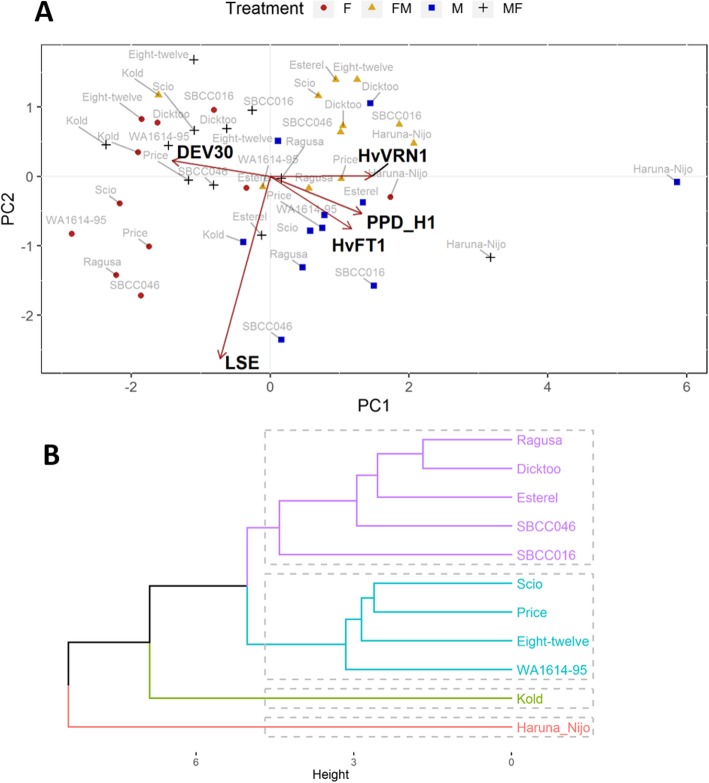
Relations between developmental phases and genetic background. **a** Principal component analysis of expression of common genes and two phases of development. Red arrows represent values of gene expression of *HvVRN1*, *HvFT1* and *PPD-H1*, and two phases of development: days from the end of vernalization to the onset of the stem elongation (DEV30), and length of the stem elongation phase (LSE). **b** Dendrogram for the 11 barley cultivars, based on phenology traits. Traits measured under 4 conditions (F, FM, MF, M)

As indicated before, most of the variation in duration of development in response to light conditions occurred before DEV30. Concurrent morphological and gene expression changes were analysed with a biplot representing a principal component analysis which included the duration of the two stages, before and after DEV30, for each genotype at each treatment, together with gene expression (Fig. 6a, excluding *HvVRN2* and *HvFT3*, which are absent in about half of the genotypes). The first and the second components explained 41.11 and 16.03% of variance, respectively (Fig. 6a). The first component clearly separates the varieties according to treatments, by overall earliness, with M and FM on the right-hand side and F on the left, with MF intermediate. This component also separates between early and late varieties overall, particularly Scio and WA1614–95, the slowest under F conditions, from Haruna Nijo, the fastest variety in all conditions. The rapid transition to the elongation phase (indicated by a low DEV30 value) was associated with high expression levels of development inducing genes *PPD-H1*, *HvVRN1* and *HvFT1* (Fig. 6a), which had large loadings with opposite signs on the first component. This was confirmed by the correlation coefficients between apex stage at the date of the RNA sampling (20–23 days after the start of the experiment) and the expression levels of *HvVRN1* (r = 0.42), *PPD-H1* (r = 0.57) and *HvFT1* (r = 0.60). The second component presented the largest loadings for the stem elongation phase, LSE (Fig. 6a). Similarly, when including all the phenophases and gene expression data in the analysis (Figure S9), DEV31 and DEV31–30 were the variables with the largest, and negative, loads on the first component, meaning that they attain higher values at the F treatment, and lower at M and FM. The second component again correlated with the various characteristics of stem elongation. In this case DEV37–30 (days from the onset of stem elongation to the appearance of the flag leaf) had the largest loading. *HvVRN2* contributed positively in this axis, indicating that its higher expression coincided with longer interval between the start of intensive stem elongation and flag leaf appearance.

In general, correlations and PCA suggested that high expression of the promoter genes of flowering favoured the acceleration of the first node appearance and the transition to the erect stage among other phenological phases, whereas the expression of *HvVRN2* was associated with lengthening of the stem elongation phase (Figure S9).

### Diversity in the response to different light sources

We carried out an exploratory analysis to classify the varieties considering their response to the four treatments, using 17 traits, which included phenological and morphological responses (Table S4). As a result, varieties were divided in 4 groups. Haruna Nijo and Kold formed one group each (Fig. 6), despite being the most insensitive varieties: Haruna Nijo was the earliest and Kold the latest across treatments. The groups were not associated with the allelic variants for the major flowering time genes, although varieties were mainly divided by geographical origin in the two main groups. Thus, varieties from USA (Price, Eight-twelve, WA1614–95 and Scio) clustered together, with the exception of Dicktoo, which was located close to Ragusa, in the European group (Ragusa, Esterel, SBCC016 and SBCC046). In general, European varieties were less influenced by light quality treatments, whereas most of the American varieties responded showing an accelerated pattern of development under metal and shift conditions.

## Discussion

### Balanced spectra accelerated plant development

Under the same light intensities, temperature and photoperiod, we observed striking differences in development that were solely due to the spectral compositions of the light sources. Fluorescent lights were rich in the green-yellow and red regions, whereas a more balanced spectra across the PAR region was produced by metal halide bulbs. Our results showed that metal halide conditions were more efficient hastening development than fluorescent light bulbs, the effect of which proved to be developmental phase specific.

In this work, we have established that light quality affects the duration of phenological phases before the onset of stem elongation, to the largest extent. The first node appearance (DEV31) and the transition between prostrate to erect state of the plant (DEV31–30) took longer to occur under fluorescent conditions. This was consistent with the dynamics of apex development, accelerated in M light, and with an increased number of leaves and longer phyllochron in F light. Therefore, the retarded growth under F light was due to a combination of having more leaves to develop, and slower rate of leaf appearance. Final number of leaves was determined early during plant development, as there was almost no difference between the two shift treatments, meaning that the conditions soon after the shift did not affect much to this trait. Phyllochron, on the other hand, was more affected by the conditions after the shift. Tillering was more profuse in fluorescent conditions although at the end of the experiment, plants grown under F showed the same number of reproductive tillers than plants in M. In addition, plants grown in F produced more spikelets per spike. The number of seeds per spike, however, was not different from the other treatments, but the seeds at F were significantly lighter. Therefore, the longer early phases under F conditions led to a longer phase of tiller production, but these plants were unable to grow all those tillers until the end and, even in that case, were less efficient in grain filling, even showing sterility or grain abortion. To the best of our knowledge, this is the first report indicating that light spectra of metal halide bulbs accelerated barley development, reducing the duration of the stem elongation phase, producing plants with less biomass and heavier seeds than in fluorescent light.

It is well known that light quality, specifically the relation of red and far-red light (R:FR ratio), is an important cue to regulate vegetative growth and the transition to reproductive stage in plants [19]. Changes in the R:FR ratio help plants detecting the presence of neighbours [20]. In response to low R:FR light ratio, many plants display a rapid and pronounced phytochrome-mediated architectural adaptation known as the shade avoidance syndrome [2, 20–22]. In shade intolerant plants, such as Arabidopsis, this syndrome consists on an increase in the elongation growth rate of stems and petioles, reduced chlorophyll content, increased apical dominance and early flowering [22, 23]. The different responses observed in this study could be related to the differences in intensity in some parts of the spectra, in particular the R:FR ratios of the systems. Fluorescent lights produced very high R:FR ratios. Metal halide bulbs also showed high ratios of R:FR compared to natural conditions (~ 1.1), but 3 times lower than fluorescent. Considering this, one could expect that plant responses under metal halide conditions could resemble those inducing shade avoidance responses. Plants grown with metal halide light showed more rapid stem elongation, flowered earlier, but had reduced number of tillers, all responses associated with shade avoidance. Yet, plant height at the end of the experiment was similar among treatments, whereas delayed apex development, extensive tillering and late flowering were observed in plants grown under fluorescent conditions. In this regard, when analysing the growth of 10 wheat cultivars under low red/far-red ratios, although plant height was unaffected, low R:FR ratios significantly reduced grain yield per plant (through grain number and, secondarily, through grain weight per plant) [1]. In our study, the comparison between M and F indicated that, on average, plants had heavier seeds with metal halide lights. All these observations suggest that even though metal halide may elicit a “shade-avoidance” response, the R:FR ratio is not low enough as to encompass a yield penalty. Other authors have already examined the effect of specific wavelengths in wheat growth and development. In a study using LED lighting, different light wavebands (blue, green and far-red) acted antagonistically over wheat development, specifically at lower intensities [3]. These authors revealed that a balanced ratio between blue and red lights accelerated flowering time in wheat. At first sight, this might agree with our results. However, the ratio B:R under metal halide and fluorescent conditions in our experiment is very similar and close to 1. Also, green light affects plant processes through cryptochrome dependent and independent mechanisms, and its effect is opposed to those driven by red and blue wavebands [24]. As fluorescent spectrum was saturated on green and red wavelengths, it could explain the delay observed in barley plants developed under this lighting regime.

### Genes and gene expression underlying light responses

As mentioned above, shade avoidance could be relevant to the responses observed. In fact, this mechanism has been described in barley, where carbon allocation in neighbouring plants was affected by changes in the red:far-red light conditions influencing the emission of volatile organic compounds [25]. Traditionally, breeding efforts have attenuated some but not all shade avoidance responses in modern crop varieties [26]. The study of phytochrome null mutants in Arabidopsis led to the confirmation that they regulate the shade-avoidance syndrome [20].

As red and blue light activate phytochromes and cryptochromes, a balanced spectrum in these regions might lead to rapid reproductive responses. Some reports also highlighted the importance of green light on developmental processes, through cryptochrome dependent mechanisms, and possibly other pathways. Its effect is generally opposed to those driven by red and blue wavebands [24]. In *Arabidopsis*, it has been reported that green wavebands have an inhibitory effect on *FT* expression, caused by direct inactivation of CRY2 protein [27], although this effect occurs mainly under low light conditions. It is worth noting that responses to green light vary among plant families, and wheat has been reported to respond to green-yellow light band (500–600 nm) by promoting earlier flowering [28]. In our experiment, fluorescent spectrum was richer on the green and red wavelengths, which could explain the delay observed in barley plants developed under this lighting regime.

The distinct spectra of fluorescent and metal halide bulbs caused differences in the expression levels of the major flowering times genes, even after full vernalization and day length of 16 h provided equally inductive conditions for all genotypes in both chambers. Positive regulators of flowering (*HvVRN1*, *PPD-H1* and *HvFT1*) showed higher transcript levels in metal halide conditions than in fluorescent conditions for most of the varieties under study (Kold was the exception, although its expression levels were probably too low to be very precise). This effect was clear and is one of the main findings of this study. Other studies have found a dependence of *FT* levels on light-quality, in this case related to the effect of different R:FR ratios on *Arabidopsis* mutants, and suggested a regulation by phyA and ELF3 [29]. As broad-spectrum light sources were used in this work, we cannot discard the involvement of other wavebands in the downregulation of flowering promoters under fluorescent conditions or the upregulation under the balanced spectra of metal halide bulbs. In fact, the upregulation of the flowering promoters was in accordance with the low levels of the repressor of flowering *HvVRN2*, in metal halide conditions. This effect on vernalization genes was unexpected, as all the genotypes were fully vernalized before being transferred to the growth chambers to start the light quality treatments.

In barley, *HvVRN2* expression is under photoperiod control, being upregulated by long days [30]. Here, we found increased *HvVRN2* mRNA level in plants growing under fluorescent conditions, which was related to the reduced levels of *HvVRN1* and the lengthening of the early phases. These results actually mimic the effect of an insufficient vernalization treatment [31, 32]. *HvFT3* expression was upregulated under fluorescent conditions, compared to other treatments. A promoter role specifically under early phases of development was proposed for *HvFT3* [33], and there is evidence pointing at its promoting effect when vernalization is not complete [31, 32]. The larger expression of *HvFT3* in F could be a compensation for the partial reversal of the vernalization effect.

In this work, we highlight the high correlation between *HvVRN1* and *PPD-H1* gene expression, explained by the similar levels found between F and MF, and between M and FM conditions. Shifting light quality conditions produced rapid changes in gene expression levels. Only 10 days after the shift between conditions, expression of both genes was adapted to the new lighting treatment. *PPD-H1* plays a key role in the sensitivity to long day conditions in barley, and works as output of the circadian clock. Recently, the alteration of the circadian clock with light quality in barley has been reported [34]. We hypothesize that *PPD-H1* and *HvVRN1* are downstream factors of clock components that have been affected by the different light spectra conditions experienced. According with this, a strong downregulation of the *PPD-1* gene, and different expression of circadian clock components were observed in wheat mutants lacking *PHYB* or *PHYC* [35]. That finding could support our hypothesis, although more study is needed.

### Mixture of spectra during different phases benefits plant growth

In general, plants subjected to shift conditions behaved closer to the metal halide control, even though MF plants passed most of the time under fluorescent conditions. We compared control and shift to determine whether 10 days in other lighting regime had effect on development. We observed that the pattern of development in FM was very similar to M. Thus, plants that started their development under fluorescent conditions were delayed, but recovered very fast. The delayed development caused by fluorescent lighting in early phases was overcome rapidly in metal halide conditions. On the contrary, when grown for 10 days in metal halide conditions and then shifted, plants did not behave as those in control fluorescent conditions. Plant development in MF was more rapid than under fluorescent conditions. Thus, 10 days in M were enough to accelerate development remarkably, which was not as severely impaired by the fluorescent spectrum as for plants grown continuously in that condition.

### Different sensitivity to light quality and its biological sense for the diversity found

There were diverse responses to lighting treatments among the varieties under study. In all the treatments, Haruna Nijo was the earliest variety, being insensitive to light quality. This genotype carries a mutation in the *Phytochrome C* gene, conferring an extremely early phenotype, both under SD and LD conditions [36, 37]. We observed a gradation of responses to light quality in the other ten genotypes studied. Further research will be needed to unravel the genetic factors underlying these different responses.

This different sensitivity to light quality conditions might have its biological significance in the plant strategies that confer different ecological behaviour to cope with competitors and environment [38]. So far, research efforts on diversity of responses to light quality has been mainly focused on different responses to low R:FR, for instance, to develop insensitive plants to the canopy of neighbours [39]. Certain tall Arabidopsis lines were less affected by low R:FR than short lines, and genotypes from lower latitudes were taller than genotypes from higher latitudes [40]. Similarly, the varieties here studied showed a different plant height dynamic associated with the different location. European varieties (Ragusa, Esterel, SBCC016 and SBCC046) and the Japanese one (Haruna Nijo) tended to be taller and showed lower responsiveness to light quality conditions than those from USA (Kold, Price, WA1614–95, Eight-Twelve, Scio and Dicktoo), which were also shorter overall. This difference in plant height probably reveals different breeding histories. Three of the European varieties were selected straight out of landraces (Ragusa, and both SBCC lines), whereas all American ones come from modern breeding programs. For this reason, it is likely that American varieties carry some semi-dwarf allele. A possible link between plant height and light sensitivity should be taken into account in further studies in this area.

## Conclusions

The characterization of light quality effects has highlighted the important influence of the spectrum on early developmental stages, revealing the onset of stem elongation as the most affected, and other downstream effects on the morphology of the plant and yield components. The fluorescent light causes a partial reversal of the outcome of the vernalization process, delaying development compared to metal halide light, and affecting grain production. There is variability among barley genotypes regarding light quality sensitivity, which could be used in the selection and development of cultivars with greater competition ability. The natural variation found in the responsiveness to light quality reinforces the need of future research in genetic control of responses to different light parameters. Based on our results, we suggest that light spectra regulate the vernalization and photoperiod genes probably through the regulation of upstream elements of signalling pathways. Whether phytochromes or cryptochromes are behind the differences found between the balanced spectra of metal halide conditions and the green and red saturated spectrum of fluorescent light bulbs, deserves further research.

## Materials and methods

### Plant material

Eleven barley cultivars were included in this study (Table 1). Kold, Price, WA1614–95, Haruna Nijo, Eight-Twelve, Scio and Dicktoo were part of the US CAP project, and were kindly provided by Professor Patrick Hayes (OSU, USA) [41, 42]. Ragusa is a Croatian landrace that was obtained from the CGN Wageningen, The Netherlands. Esterel is a French cultivar from Secobra. SBCC016 and SBCC046 are two Spanish landrace-derived inbred lines from the SBCC [43]. All cultivars were multiplied in isolation at the MTA-ATK, collected from bagged spikes, from original seed provided by OSU or the group at EEAD-CSIC. The varieties have different allelic constitution in the major flowering time genes, related to vernalization and photoperiod pathways (*HvVRN1*, *HvVRN2*, *HvFT1*, *PPD-H1*, *HvFT3*), as described in Table 1. Plants were fully vernalized (5 ± 2 °C for 52 days under 8 h light/16 h night) prior to the start of the experiment. Then, plants were moved to independent growth chambers with different light bulbs, fluorescent or metal halide. All of them were established under long day photoperiod conditions (16 h light/8 h night), constant temperature of 18 ± 1 °C during day and night, and the same light intensity (~ 250 μmol m^− 2^ s^− 1^).

### Light spectral conditions

The experiments were carried out in the Phytotron facilities of the Agricultural Research Institute of the Hungarian Academy of Sciences, at Martonvásár (Hungary) using Conviron PGR-15 growth chambers (Conviron Ltd., Canada).

Two broad-spectrum lamp types with major differences within the photosynthetically active region (PAR, 400–700 nm) were used: Sylvania cool white fluorescent (F) and Tungsram HGL-400 metal halide (M) light bulbs. Absolute photon irradiance was obtained with a USB400-UV-VIS Spectrometer (Ocean Optics, USA), measuring PAR at the top of the plant canopy. Spectral data were based on 0.21 nm intervals from 350 to 873 nm, calculated as spectral photon distribution and the summation over the interval as photon flux [44]. Spectral energy distribution of light, in both light sources, was studied in the 400–850 nm wavelength region (Figure S1A). The ratios between the photon flux densities at different wavelengths are given in Figure S1B (following Mortensen and Stromme [45]). Blue region (B) in the range 400–500 nm; green-yellow (GY), 500–600 nm; red (R), 600–700 nm; far-red (FR), 700–800 nm. Major differences within the PAR region are due to green-yellow-red ratio (GY:R) and red-far-red ratio (R:FR) (Figure S1).

### Comparison between light quality treatments

The study consisted in two control treatments and two shift treatments, grown in two growth chambers, each equipped with a different lighting type. The shift treatments were carried out first and, when these were finished, the two control treatments were performed. For the control treatments, the plants were kept at the same growth chamber (M or F) for the full duration of the experiment. For the shift treatments, plants were moved to one of the chambers after vernalization and, 10 days later, the entire sets of plants were interchanged (M plants moved to F chamber and vice versa). The shift treatments are coded as MF and FM. For phenotypic measurements, sixteen plants per genotype were sown in individual pots, and four were placed at each growth chamber, making four replicates per genotype and treatment. Additionally, 20 seeds per genotype and treatment were sown in groups of 5 plants/pot, which were used for destructive samplings to record apex development stage and for gene expression studies.

### Phenotypic measurements

Plant development was monitored twice a week by counting leaf and tiller number, measuring plant height, and checking for first node appearance (plant developmental stage 31, or DEV31) and appearance of the awns just visible above the last leaf sheath (DEV49). All these data were used to dissect the development in phenophases, defined based on stages of the Zadoks’ scale [46], following the description of Tottman et al. [47]. Dynamics of development was measured following Kiss et al. [48]. DEV30 denotes the start of the elongation phase, and DEV37 and DEV39 are the days to the appearance and full elongation of the flag leaf, respectively. Another calculated trait was the time to reach the 50% of the plant height (PH_50_).

In control conditions, apex dissection was carried out 8, 23, 31, 37 and 45 days after the end of the vernalization period. In the shift conditions, apex dissection was carried out 20, 30 and 41 days after the vernalization period. At each sampling point, 3 plants per variety and treatment were dissected. Phenotyping consisted on recording apex length (mm) and apex stage following the Waddington’s scale [49]. For gene expression analysis, the last fully expanded leaf was collected in the middle of the light cycle at each particular treatment, 20 days after the end of the vernalization period, in three plants per genotype and sampling.

The plants were grown to full maturity and the following yield components were evaluated: number of nodes in the main stem, length of the last internode in the main stem (cm), spike length in the main stem (cm), number of spikelets per spike in the main stem, number of fertile tillers (reproductive tillers), number of seeds in the main stem, individual seed weight in the main stem (g), total number of seeds per plant, and total seed weight in the whole plant (g).

### Gene expression

Total RNA was isolated from leaf tissue with TRIzol Reagent (Thermo Fisher Scientific, Ltd) followed by the Qiagen RNeasy Plant Mini kit, in accordance with the manufacturer instructions (Qiagen, Ltd.). Then, the material was extracted in the QIAcube equipment (Qiagen Ltd). One microgram of total RNA was used for cDNA synthesis using the Revert Aid First Strand cDNA synthesis kit (Thermo Fisher Scientific, Ltd) with the standard protocol provided by the company. The quantitative real time PCR was carried out in three biological and two technical replicates in a Rotor-Gene Q equipment (Qiagen Ltd) with SYBR-Green Master Mix. Primer sequences of the genes evaluated are described in Table S5. Expression was normalized to *Actin* using the Rotor-Gene software, also accounting for the amplification efficiency.

### Statistical analysis

Data from 11 varieties growing in four different treatments (MF, FM, M, F) were used in the analyses, taking into account the average of 3–4 biological replicates. In the representation of the dynamics of the apex stages, 95% confidence interval was calculated using a local polynomial regression model. All the analyses were carried out in R [50]. Analysis of variance were performed considering all the factors (genotypes and treatments) as fixed, and replicates nested within treatments. When comparing groups of genotypes, contrasts were calculated within the factor genotype and in its interaction with treatment. To test their significance, we calculated the ratio of the mean squares of each contrast to the mean squares corresponding to the pooled within genotype variance not explained by each contrast. Multiple comparisons were obtained by Fisher’s protected Least Significant Differences (LSD) with the R package ‘agricolae’ [51]. Pearson correlations were obtained using the package ‘corrplot’ [52]. Principal component analysis (PCA) was performed with the R function ‘prcomp’ using the singular value decomposition, and based on the correlation matrix. Biplot was carried out with the package ‘ggplot2’ [53]. To assess the phenotypic diversity through a cluster analysis, all variables in common in all the treatments were taken into account, and highly related variables were discarded. Finally, 17 phenotypic traits were used to carry out the cluster analysis (Table S4). Cluster analysis (UPGMA) was done with the package “factoextra” [54]. The best grouping result was chosen as the one with the highest cophenetic correlation and the lowest Gower distance.

## Supplementary information

**Additional file 1.** Supplementary data

## Data Availability

The datasets used and/or analysed during the current study available from the corresponding author on reasonable request.

## Abbreviations

B: Blue region
DEV: Developmental stage
F: Fluorescent
FM: Shift from fluorescent to metal halide conditions
FR: Far-red region
GY: Green-yellow region
M: Metal halide
MF: Shift from metal halide to fluorescent conditions
PAR: Photosynthetically active region
R: Red region
