# Supplementary material for: Genetic diversity in developmental responses to light spectral quality in barley (Hordeum vulgare L.)
Source: BMC Plant Biol. 2020 May 12;20:207. doi: 10.1186/s12870-020-02416-1 (PMC7216675; doi:10.1186/s12870-020-02416-1)
Supplement: Supplementary file 1 — Additional file 1. Supplementary data [file 12870_2020_2416_MOESM1_ESM.docx]

# Supplementary data

**Genetic diversity in developmental responses to light spectral quality in barley (*Hordeum vulgare* L.)**

Arantxa Monteagudo, Tibor Kiss, Marianna Mayer, Ana M. Casas, Ernesto Igartua, Ildikó Karsai

# Supplementary data

Supplementary Table S1. Analysis of variance for Waddington stage after 30 days from the end of the vernalization.

Supplementary Table S2. Analysis of variance and comparison between groups of varieties and genotypes.

Supplementary Table S3. Multiple comparisons of gene expression.

Supplementary Table S4. Description of traits used for cluster analyses.

Supplementary Table S5. Primer sequences for gene expression assay.

Supplementary Figure S1. Spectral composition of fluorescent (F) and metal halide (M) light bulbs.

Supplementary Figure S2. Analyses of variance for DEV31, DEV30, length of stem elongation (LSE) and DEV49. days to 50% of plant height (PH50) and final plant height (PH).

Supplementary Figure S3. Duration of phenophases in each variety and treatment.

Supplementary Figure S4. Analyses of variance for phyllochron (days^-1^), final leaf number, days to 50% of plant height (PH50) and final plant height (PH).

Supplementary Figure S5. Comparison of days to first node appearance (DEV31) in fluorescent versus metal halide conditions.

Supplementary Figure S6. Dynamics of apex length and morphology in the shift treatments.

Supplementary Figure S7. Dynamic of the number of leaves under different light quality conditions.

Supplementary Figure S8. Dynamics of the number of tillers under different light quality conditions.

Supplementary Figure S9. Relations between phenophases and expression of flowering time genes.

**Table S1.** Analysis of variance for Waddington stage 30 days after the end of the vernalization. Df, degrees of freedom.

| Source of variation | Df | Sums of squares | Mean squares | Variance ratio | F probability |
| --- | --- | --- | --- | --- | --- |
| *Variety* | 10 | 400.4 | 40.0 | 205.5 | <.001 |
| *Treatment* | 3 | 264.9 | 88.3 | 453.3 | <.001 |
| *Replicate: Treatment* | 8 | 1.6 | 0.2 | 1.0 | 0.414 |
| *Variety.Treatment* | 30 | 105.0 | 3.5 | 18.0 | <.001 |
| *Residual* | 76 | 14.8 | 0.2 |  |  |

‘:’ denotes ‘nested to’. ‘.’ Denotes ‘interaction between’.

**Table S2.** Analysis of variance and comparison between groups of varieties and genotypes.

|  |  | **DEV31** | | **DEV30** | | **LSE** | | **DEV49** | | **PH_final_** | |
| --- | --- | --- | --- | --- | --- | --- | --- | --- | --- | --- | --- |
| Source of variation | df | Mean squares | F probability | Mean squares | F probability | Mean squares | F probability | Mean squares | F probability | Mean squares | F probability |
| *Variety* | 10 | 470 | <.001 | 1,472 | <.001 | 37 | <.001 | 1,621 | <.001 | 1,467 | <.001 |
| *Sensitive vs. Insensitive^a^* | 1 | 1,994 | 0.030 | 1,721 | 0.303 | 6 | 0.699 | 2,073 | 0.280 | 8,433 | 0.007 |
| *HvVRN1 vs. Hvvrn1* | 1 | 87 | 0.690 | 0 | 1.000 | 1 | 0.885 | 18 | 0.923 | 109 | 0.801 |
| *HvVRN2 vs. Hvvrn2* | 1 | 755 | 0.222 | 4,932 | 0.062 | 7 | 0.682 | 4,811 | 0.083 | 2 | 0.973 |
| *HvFT3 vs. Hvft3* | 1 | 273 | 0.475 | 24 | 0.906 | 102 | 0.097 | 25 | 0.909 | 253 | 0.700 |
| *HvFT1(AG) vs. HvFT1(TC)* | 1 | 424 | 0.369 | 1,368 | 0.362 | 0 | 0.923 | 1,876 | 0.306 | 363 | 0.644 |
| *Residual (per contrast)^b^* | 9 |  |  |  |  |  |  |  |  |  |  |
| *Treatment* | 3 | 1,269 | <.001 | 6,444 | <.001 | 132 | <.001 | 8,893 | <.001 | 104 | <.001 |
| *Replicate: Treatment* | 12 | 4 | 0.311 | 3 | 0.796 | 6 | 0.428 | 2 | 0.57 | 6 | 0.784 |
| *Variety.Treatment* | 30 | 57 | <.001 | 135 | <.001 | 9 | 0.086 | 149 | <.001 | 43 | <.001 |
| *Sensitive vs. Insensitive* | 3 | 138 | 0.093 | 210 | 0.243 | 5 | 0.685 | 246 | 0.219 | 2 | 0.988 |
| *HvVRN1 vs. Hvvrn1* | 3 | 62 | 0.401 | 104 | 0.554 | 2 | 0.919 | 168 | 0.385 | 9 | 0.902 |
| *HvVRN2 vs. Hvvrn2* | 3 | 123 | 0.125 | 331 | 0.090 | 1 | 0.964 | 308 | 0.138 | 49 | 0.380 |
| *HvFT3 vs. Hvft3* | 3 | 90 | 0.237 | 249 | 0.177 | 6 | 0.577 | 297 | 0.150 | 21 | 0.722 |
| *HvFT1(AG) vs. HvFT1(TC)* | 3 | 20 | 0.811 | 55 | 0.774 | 14 | 0.228 | 122 | 0.528 | 206 | 0.006 |
| *Residual (per contrast)^b^* | 27 |  |  |  |  |  |  |  |  |  |  |
| *Residual* | 119 | 3 |  | 5 |  | 6.1 |  | 3 |  | 9 |  |

*^a^ Sensitive vs. insensitive*, as defined in Figure 6B.

*^b^* Residuals calculated independently for each contrast, as the *Variety* sums of squares not explained by the contrast, and the *Variety.Treatment* sums of squares not explained by each contrast, respectively

**Table S3.** Multiple comparisons of gene expression. For each expression point in figure 6, data with the same letter are not significantly different at P < 0.05 according to ANOVA that included genotypes and treatments.

|  | ***HvVRN1*** | | | | ***PPD-H1*** | | | | ***HvFT1*** | | | | ***HvFT3*** | | | | ***HvVRN2*** | | | |
| --- | --- | --- | --- | --- | --- | --- | --- | --- | --- | --- | --- | --- | --- | --- | --- | --- | --- | --- | --- | --- |
| **Variety/Treatment** | MF | F | FM | M | MF | F | FM | M | MF | F | FM | M | MF | F | FM | M | MF | F | FM | M |
| Dicktoo | b | b | a | a | c | bc | b | a | a | b | a | a | ND | | | | ND | | | |
| Eight-twelve | b | b | a | b | b | b | a | a | b | b | a | b | b | a | b | b | b | b | b | a |
| Esterel | b | b | ab | a | c | ab | b | a | a | b | ab | a | ND | | | | b | b | a | ab |
| Haruna-Nijo | b | b | b | a | b | b | b | a | a | c | c | b | b | a | b | b | ND | | | |
| Kold | b | a | ab | ab | b | b | b | a | b | a | b | b | ND | | | | bc | c | b | a |
| Price | b | b | a | a | b | b | a | a | b | b | a | a | b | a | b | b | b | a | b | b |
| Ragusa | ab | b | a | a | a | a | a | a | b | b | a | a | ND | | | | ND | | | |
| SBCC016 | c | c | a | b | c | c | b | a | b | b | b | a | b | a | b | b | b | a | b | b |
| SBCC046 | b | b | a | b | c | c | b | a | bc | c | b | a | ND | | | | b | a | b | b |
| Scio | bc | b | ab | a | b | b | a | a | c | c | b | a | c | c | b | a | ND | | | |
| WA1614-95 | bc | c | b | a | b | b | a | a | b | b | a | a | ND | | | | ND | | | |

ND, not detected.

**Table S4.** Description of traits used for cluster analyses.

|  | **Variable** | **Description** |
| --- | --- | --- |
| 1 | DEV31 | Days* to the first node appearance |
| 2 | DEV49 | Days* to awns appearance |
| 3 | FLN | Final leaves number |
| 4 | LFdev31 | Leaves number at DEV31 |
| 5 | PhyllDays | Phyllochron (leaves days^-1^) |
| 6 | PhFinal | Final plant height |
| 7 | LSE | Length (days) of stem elongation phase |
| 8 | PH49 | Plant height at DEV49 |
| 9 | PH31 | Plant height at DEV31 |
| 10 | SGdays | Stem growth ratio (cm days^-1^) |
| 11 | DEV30 | Days* to onset of stem elongation |
| 12 | DEV37 | Days* to initiation of flag leaf |
| 13 | DEV39 | Days* to complete flag leaf formation |
| 14 | ZDSE | Days* to end of stem elongation |
| 15 | Spike_length | Spike length in the main stem at harvest |
| 16 | Spikelet_number | Number of spikelets (triplets) in the main stem’s spike at harvest |
| 17 | Rep_Till | Number of fertile side tillers in addition to the main spike at harvest |

*Days from the end of vernalization.

**Table S5**. Primer sequences for gene expression assay. F, primer forward; R, primer reverse.

| **ID** | **PRIMER SEQUENCE (5'-3')** | **Reference** |
| --- | --- | --- |
| *HvVRN1* | F: TATGAGCGCTACTCTTATGC  R: TGAAGCTCAGAAATGGATTCG | Trevaskis *et al.* (2006) |
| *HvVRN2* | F: GAGCCACCATCGTGCCATTC  R: GCCGCTTCTTCCTCTTCTC | Trevaskis *et al.* (2006) |
| *HvFT1* | F: ATCTCCACTGGTTGGTGACAGA  R: TTGTAGAGCTCGGCAAAGTCC | Yan *et al.* (2006) |
| *PPD-H1* | F: CAAATCAAAGAGCGGCGATC  R: TCTGACTTGGGATGGTTCACA | Hemming *et al.* (2008) |
| *HvFT3* | F: GGTTGTGGCTCATGTTATGC  R: CTACTCCCCTTGAGAACTTTC | Forward: Kikuchi *et al.* (2009); Reverse: Faure *et al. (*2007) |
| *Actin* | F: GCCGTGCTTTCCCTCTATG  R: GCTTCTCCTTGATGTCCCTTA | Trevaskis *et al.* (2006) |


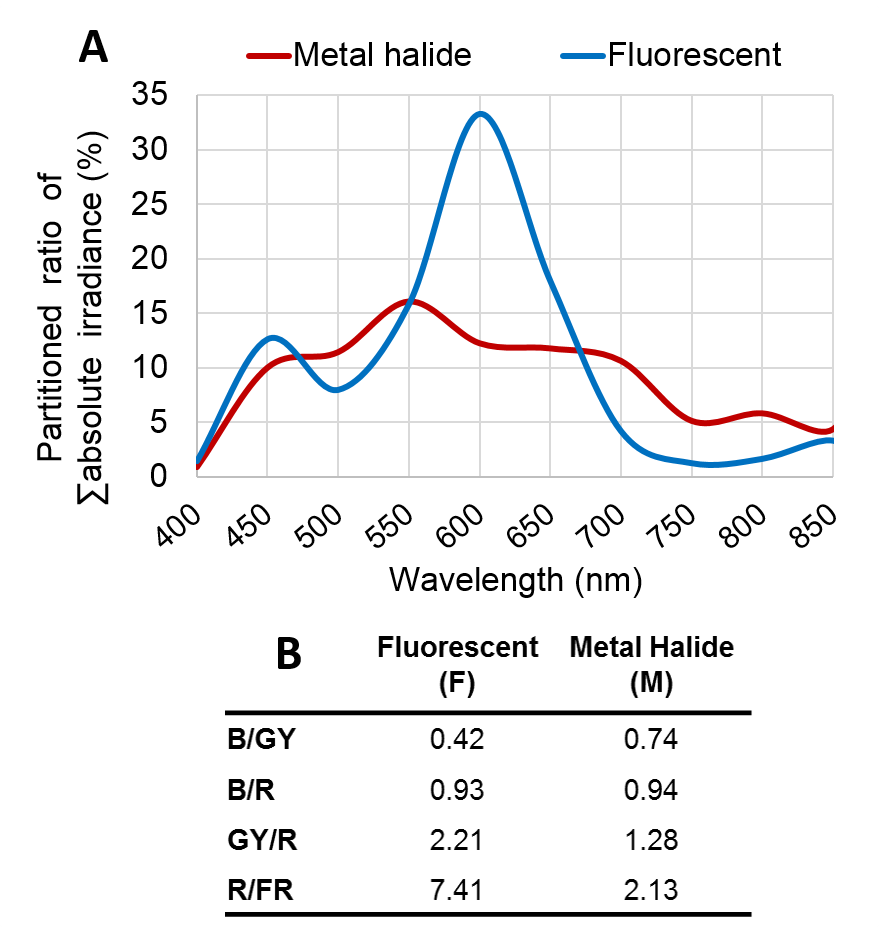


**Figure S1.** Spectral composition of fluorescent (F) and metal halide (M) light bulbs. A) Proportion of irradiance (%) measured in the growth chambers. B) Ratios between different wavebands. Ratio measured as photon flux density. B, blue (400-500 nm); GY, green-yellow (500-600 nm); R, red (600-700 nm); FR, far-red (700-800 nm).


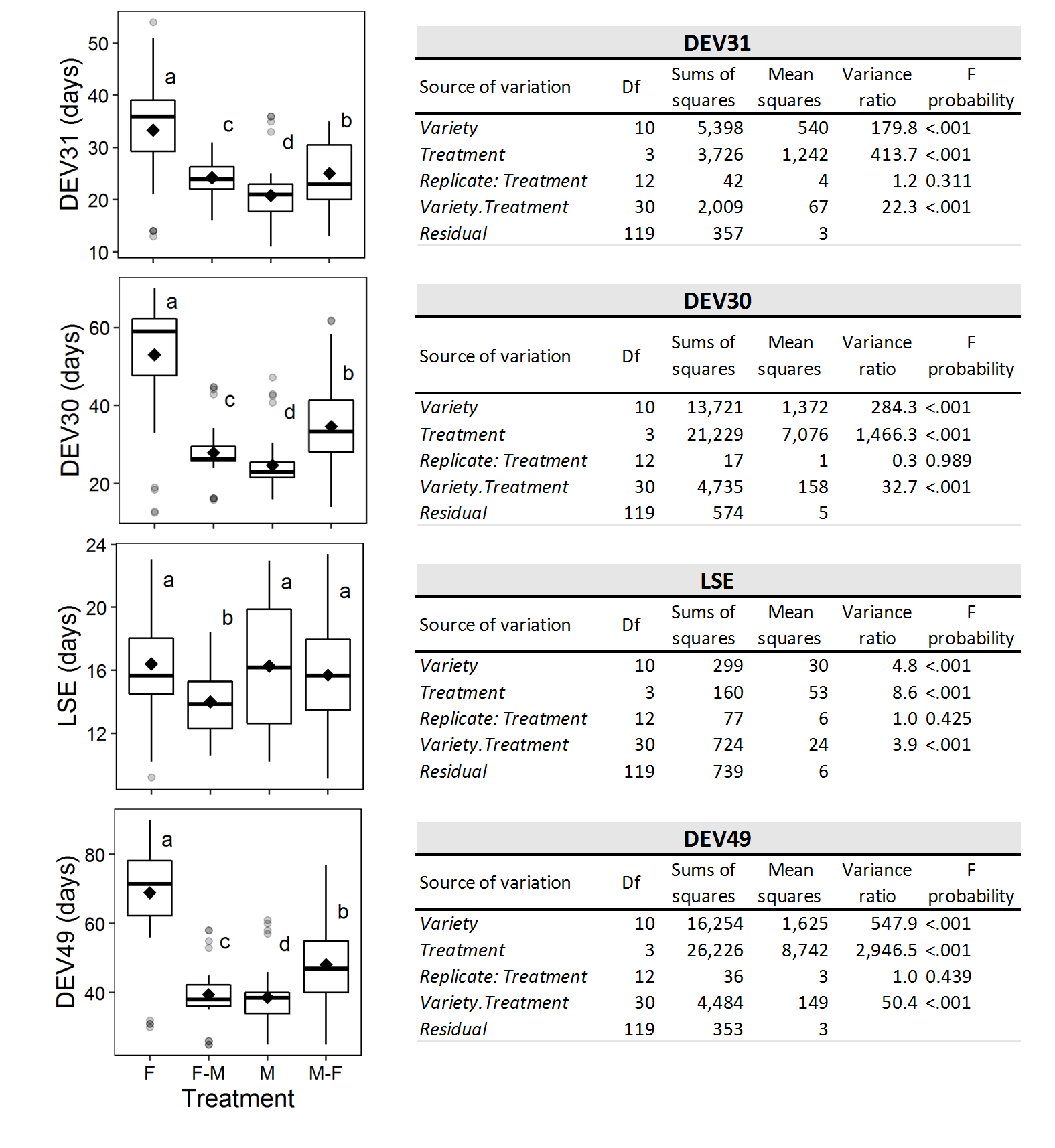


**Figure S2.** Boxplot of DEV31, DEV30, length of stem elongation (LSE) and DEV49, and analysis of variance of each variable. Results derived from 4 plants per variety, 11 varieties. In each boxplot, the mean is represented with a black diamond; the horizontal bar splitting the box represents the median; the height of the box represents the interquartile range, and the whiskers length represent 1.5 times the interquartile range. Different letters represent significant differences between treatments in a post-hoc LSD-test, for a P-value <0.05. In the ANOVA, all the factors are considered fixed. ‘:’ indicates “nested to” and “.” indicates “interaction”.


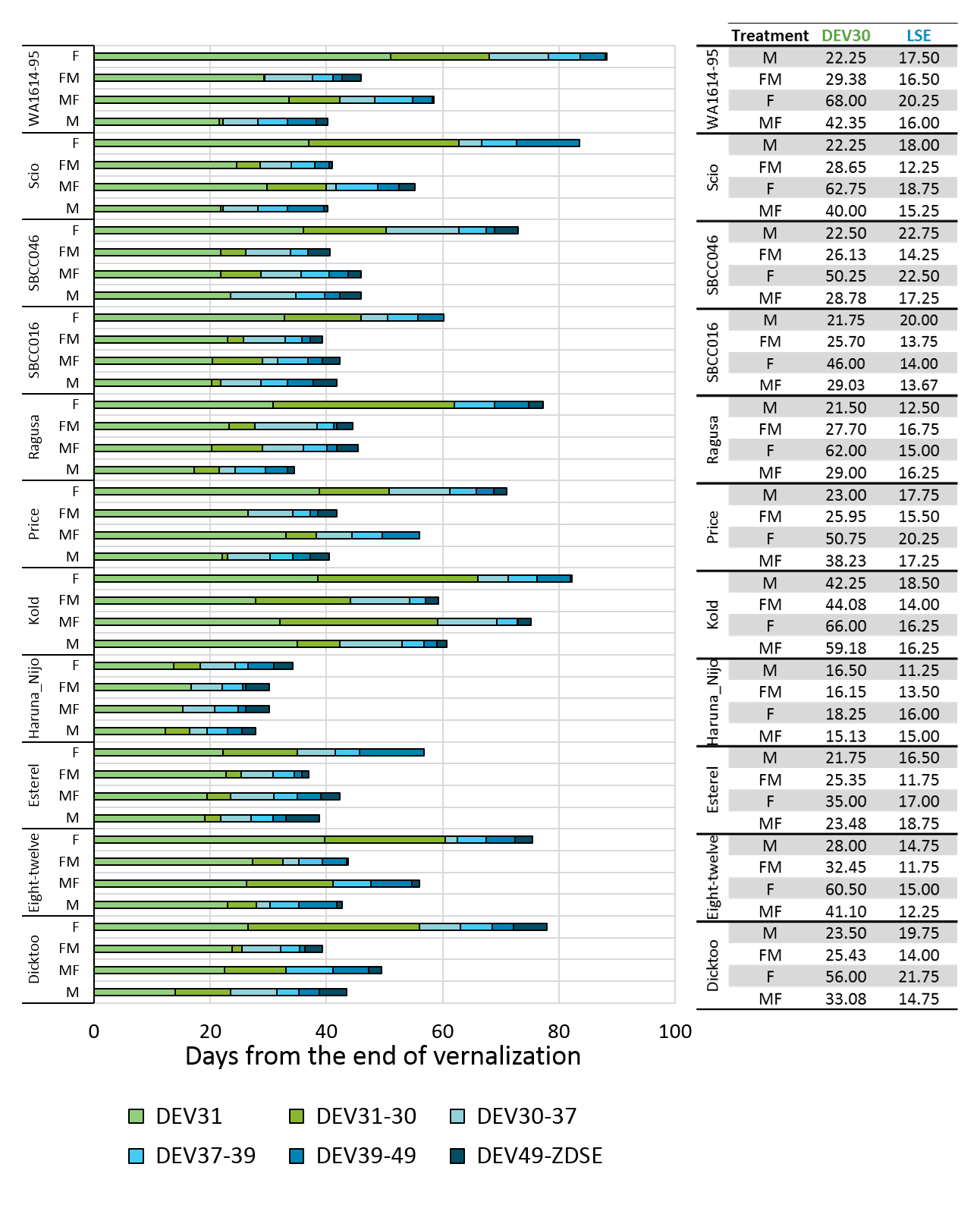


**Figure S3**. Duration of phenophases in each variety and treatment. DEV31, first node appearance; DEV31-30, days from the first node appearance to the onset of the stem elongation; DEV30-37, days from the onset of the stem elongation to the appearance of the flag leaf; DEV37-39, days to complete expansion of the flag leaf; DEV39-DEV49, days from the completion of the flag leaf to the awns appearance; and DEV49-ZDSE, days from awns appearance to the end of the stem elongation. Table represents the days from the end of vernalization to the onset of stem elongation (DEV30), which comprises DEV31 and DEV30, and the length of the stem elongation phase (LSE), as days from DEV30 to the end of stem elongation (ZDSE). Mean of 3-4 biological replicates is represented.


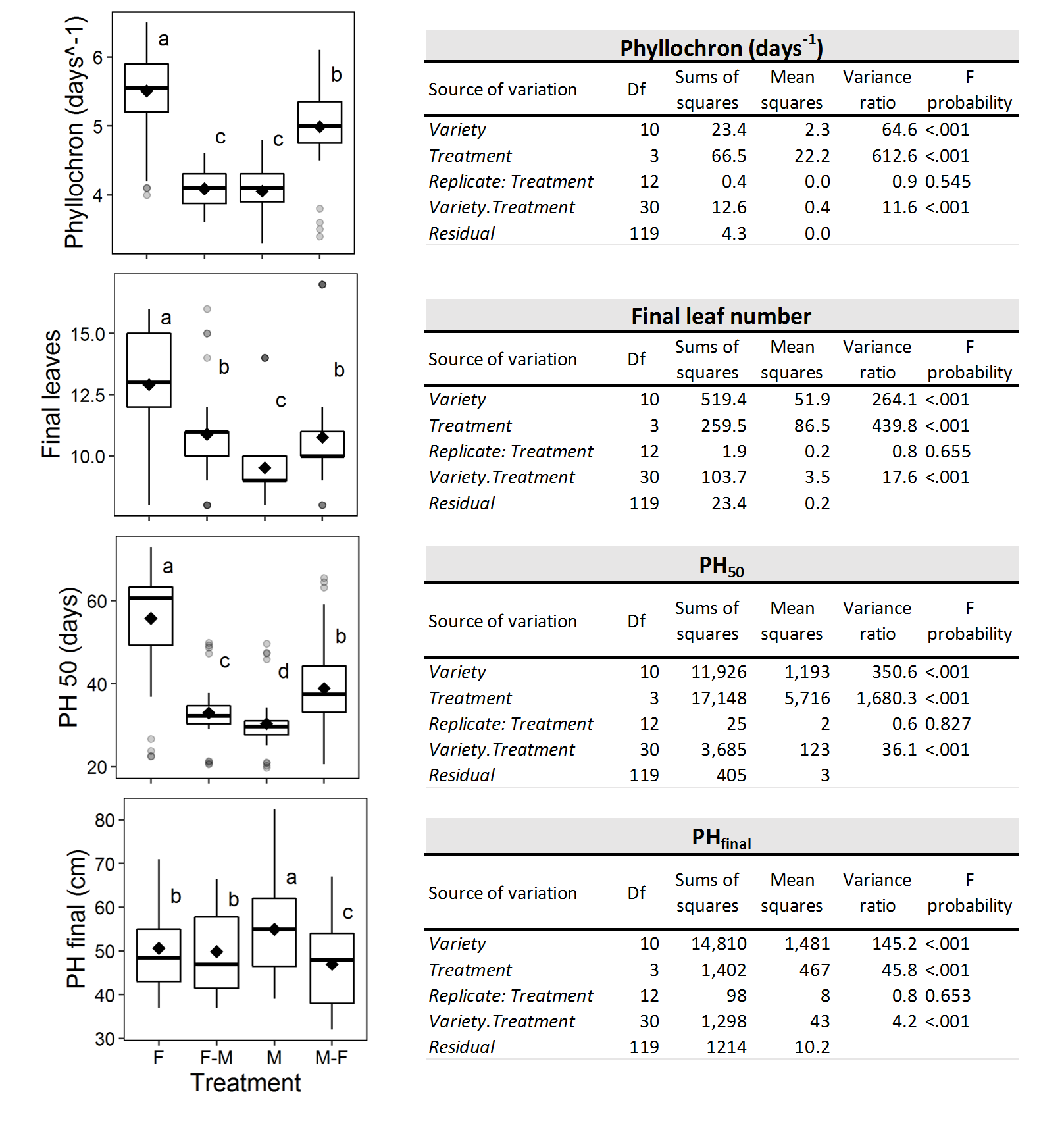


**Figure S4.** Boxplot of phyllochron (days^-1^), final leaf number in the main stem, days to the 50% of final plant height (PH50) and final plant height (PH final), and analysis of variance of each variable. Results derived from 4 plants per variety, 11 varieties. In each boxplot, the mean is represented with a black diamond; the horizontal bar splitting the box represents the median; the height of the box represents the interquartile range, and the whiskers length represent 1.5 times the interquartile range. Different letters represent significant differences between treatments in a post-hoc LSD-test, for a P-value <0.05. In the ANOVA, all the factors are considered fixed.‘:’ indicates “nested to” and “.” indicates “interaction”.


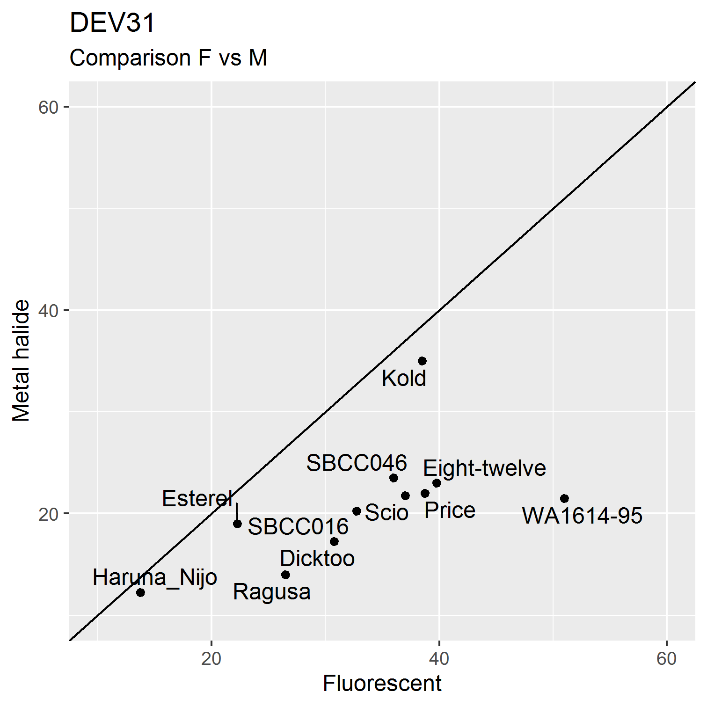


**Figure S5.** Comparison of days to first node appearance (DEV31) in fluorescent versus metal halide conditions. Each dot represents the average of 4 biological replicates per variety. The diagonal denotes the 1:1 line.


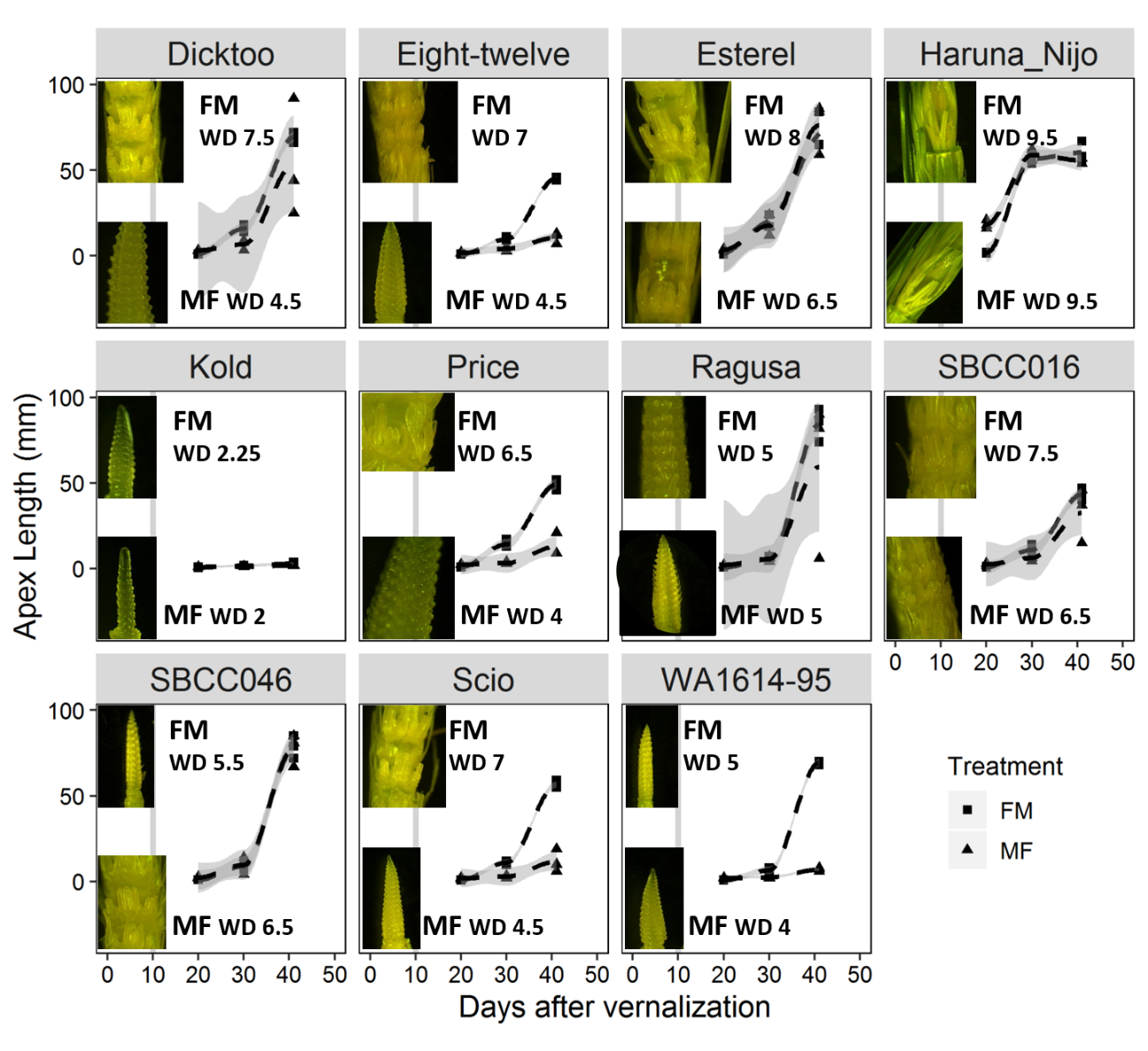


**Figure S6.** Dynamics of apex length and morphology in the shift treatments. Triangles represent MF and rectangles, FM. The vertical solid grey line denotes the shift day (the day when plants were switched between growth chambers with different light quality treatments). The shaded area indicates the 95% confidence interval (loess smooth line) calculated using a polynomial regression model. Apex photos were taken 30 days after the end of the vernalization.


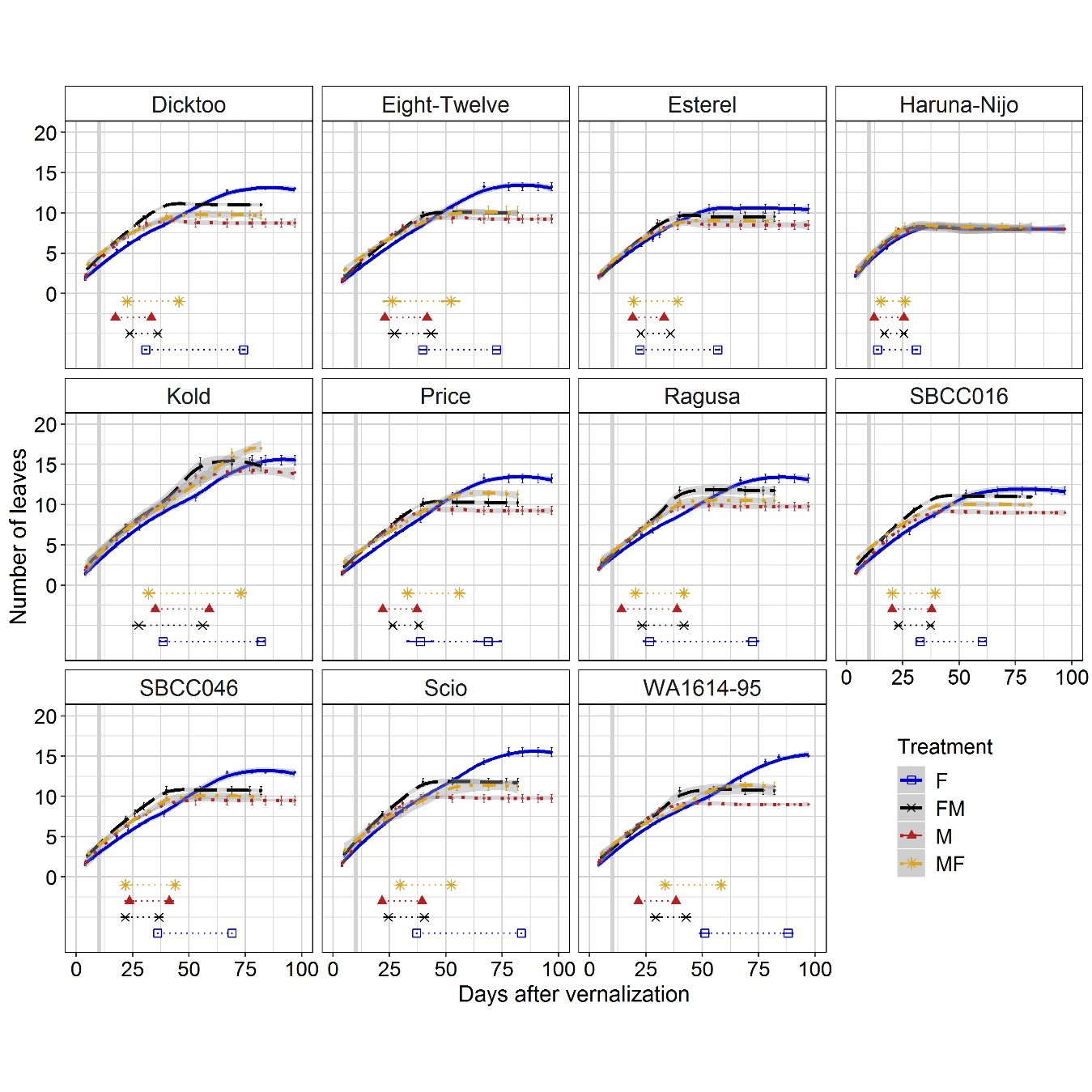


**Figure S7.** Dynamic of the number of leaves under different light quality conditions. Each block represents a variety. Solid line, fluorescent; dashed line, FM; dotted line, M; dot-dashed line, MF. Each line represents the average of 4 biological replicates. The shaded area indicates the 95% confidence interval (Loess smooth line) calculated using a polynomial regression model. Under the curves, horizontal lines represent the time from first node appearance (DEV31, first dot) to awns appearance (DEV49) in the main stem. Vertical solid grey line denotes the shift day: the day when light quality treatments were changed.


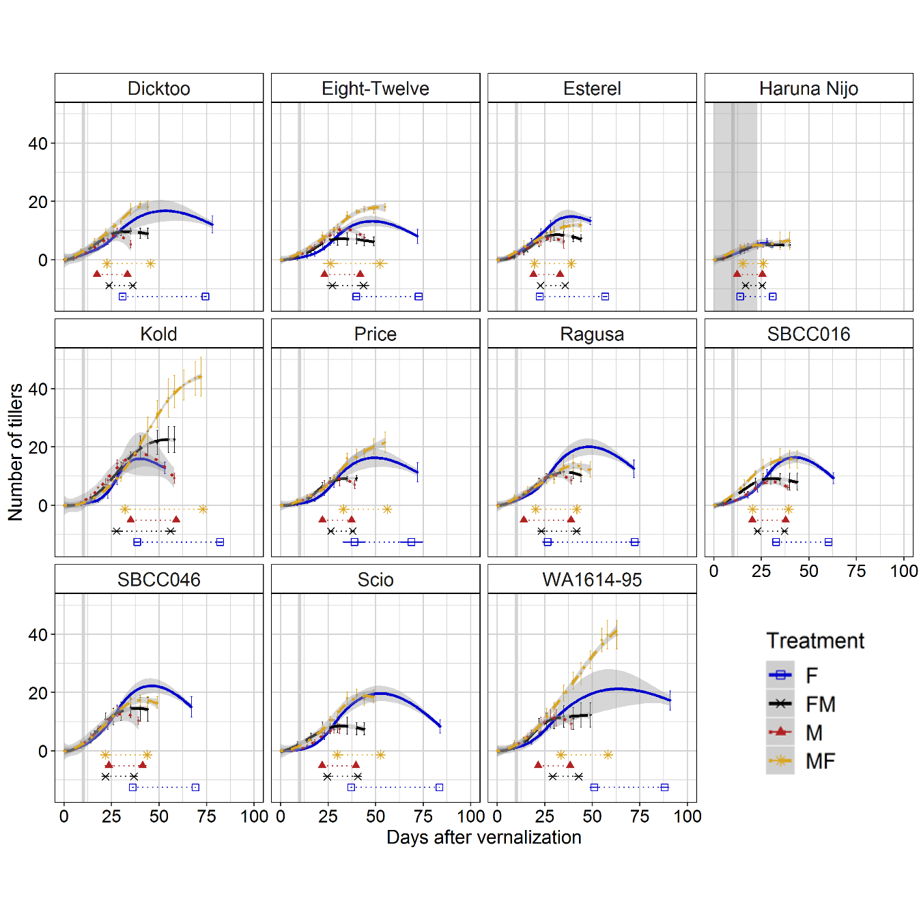


**Figure S8**. Dynamics of the number of tillers under different light quality conditions. Each block represents a variety. Solid line, fluorescent; dashed line, FM; dotted line, M; dot-dashed line, MF. Each line represents the average of 4 biological replicates. The shaded area indicates the 95% confidence interval (Loess smooth line) calculated using a polynomial regression model. Under the curves, horizontal lines represent the time from first node appearance (DEV31, first dot) to awns appearance (DEV49) in the main stem. Vertical solid grey line denotes the shift day: the day when light quality treatments were changed.


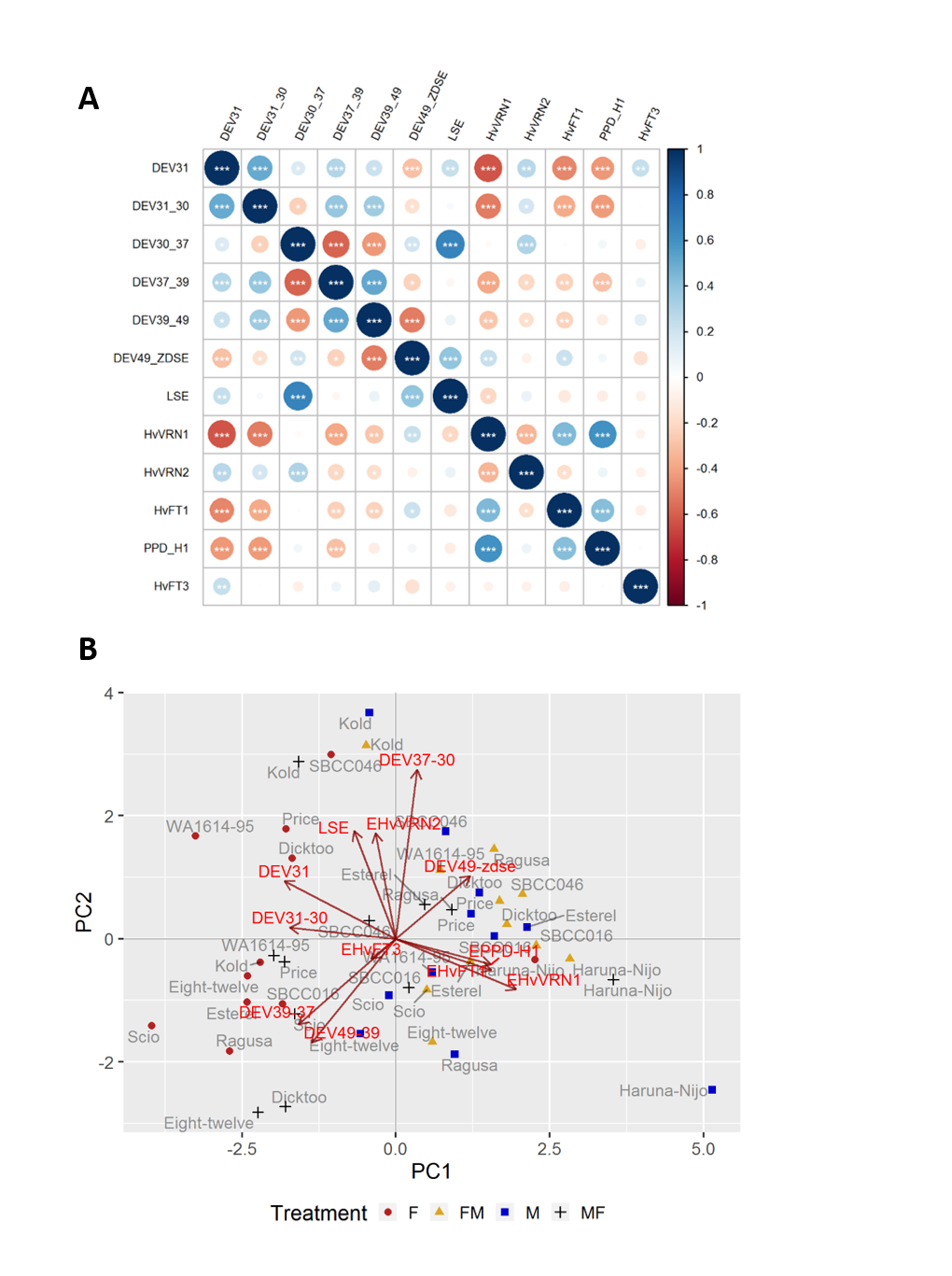


**Figure S9** Relations between phenophases and expression of flowering time genes. A) Pearson correlations, taking into account the averages of 11 varieties in 4 light quality treatments (for phenophases, n=4; for gene expression, n=3). B) Biplot for the principal component analysis based on the correlation matrix for all four treatments combined. PC1 and PC2 explain 32.64% and 19.43% of the variance, respectively.

**References**

Faure S, Higgins J, Turner A, Laurie DA. The *FLOWERING LOCUS T*-like gene family in barley (*Hordeum vulgare*). Genetics. 2007;176:599–609.

Hemming MN, Peacock WJ, Dennis ES, Trevaskis B. Low-temperature and daylength cues are integrated to regulate FLOWERING LOCUS T in barley. Plant Physiol. 2008;147:355–66.

Kikuchi R, Kawahigashi H, Ando T, Tonooka T, Handa H. Molecular and functional characterization of PEBP genes in barley reveal the diversification of their roles in flowering. Plant Physiol. 2009;149:1341–53

Trevaskis B, Hemming MN, Peacock WJ, Dennis ES. *HvVRN2* responds to daylength, whereas *HvVRN1* is regulated by vernalization and developmental status. Plant Physiol. 2006;140:1397–405.
